# Gastric microbiota: an emerging player in gastric cancer

**DOI:** 10.3389/fmicb.2023.1130001

**Published:** 2023-04-27

**Authors:** Shizhen Zhou, Chenxi Li, Lixiang Liu, Qinggang Yuan, Ji Miao, Hao Wang, Chao Ding, Wenxian Guan

**Affiliations:** ^1^Department of General Surgery, Nanjing Drum Tower Hospital, The Affiliated Hospital of Nanjing University Medical School, Nanjing, Jiangsu, China; ^2^Laboratory Medicine Center, The Second Affiliated Hospital of Nanjing Medical University, Nanjing, Jiangsu, China; ^3^Department of General Surgery, Nanjing Drum Tower Hospital Clinical College of Nanjing Medical University, Nanjing, Jiangsu, China; ^4^Department of General Surgery, Nanjing Drum Tower Hospital Clinical College of Xuzhou Medical University, Nanjing, Jiangsu, China

**Keywords:** gastric cancer, *H. pylori*, gastric microbiota, non-*H. pylori*, microbiome diversity

## Abstract

Gastric cancer (GC) is a common cancer worldwide with a high mortality rate. Many microbial factors influence GC, of which the most widely accepted one is *Helicobacter pylori* (*H. pylori*) infection. *H. pylori* causes inflammation, immune reactions and activation of multiple signaling pathways, leading to acid deficiency, epithelial atrophy, dysplasia and ultimately GC. It has been proved that complex microbial populations exist in the human stomach. *H. pylori* can affect the abundance and diversity of other bacteria. The interactions among gastric microbiota are collectively implicated in the onset of GC. Certain intervention strategies may regulate gastric homeostasis and mitigate gastric disorders. Probiotics, dietary fiber, and microbiota transplantation can potentially restore healthy microbiota. In this review, we elucidate the specific role of the gastric microbiota in GC and hope these data can facilitate the development of effective prevention and therapeutic approaches for GC.

## Introduction

Gastric cancer (GC) ranks fifth most common and third most deadly cancer globally (Rawla and Barsouk, 2019). Factors that induce gastric carcinogenesis include gastric microbiota, alcohol, smoking, and unhealthy dietary (Dong and Thrift, 2017; Zhao et al., 2017). Among many risk factors for GC, gastric microbiota act as an emerging one. Human gastric microbiota are subject-specific species and include a variety of bacteria. *H. pylori* is classified as a Class I risk factor for GC by the World Health Organization (WHO), and *H. pylori* infection is widely regarded as the strongest threat to GC (Wroblewski et al., 2010). *H. pylori* has a high infection rate and frequently colonized more than half of the world’s population. The infection of *H. pylori* usually occurs during childhood and will last for a lifetime (Malaty et al., 2002). *H. pylori* can disturb the human immune system and promote inflammation responses, leading to acid deficiency, epithelial atrophy, and dysplasia (Doorakkers et al., 2016). Diverse species more common than *H. pylori* have been found in gastric samples, such as *Streptococcus*, *Prevotella*, *Veronella*, *Clostridium*, *Haemophilus*, and *Neisseria* (Rajilic-Stojanovic et al., 2020). These gastrointestinal microbiota exhibit different biological functions, for instance, preventing the invasion of pathogens, digesting complex carbohydrates, regulating immune response, or regulating the central nervous system (Alarcón et al., 2017).

The process for analyzing the diversity of the gastric microbiota has undergone a change from culture-based methods to molecular assays. Early studies relied on culture-based analysis (Wang et al., 2020a). And the emergence of next-generation sequencing (NGS) enabled researchers to analyze the composition and function of microbiota in a diverse environment with higher throughput and resolution, mainly including targeted amplicon sequencing by 16S ribosomal RNA (rRNA) genes and shotgun metagenomics (Boers et al., 2019), providing fascinating insights into the human gastric microbiota. In this review, we mainly analyzed the basic composition of microbiota in the human stomach, illustrated the changes and interactions of gastric microbiota in GC, and discussed promising strategies to regulate gastric microbiota.

## Composition of the gastric microbiota

**Figure 1:**
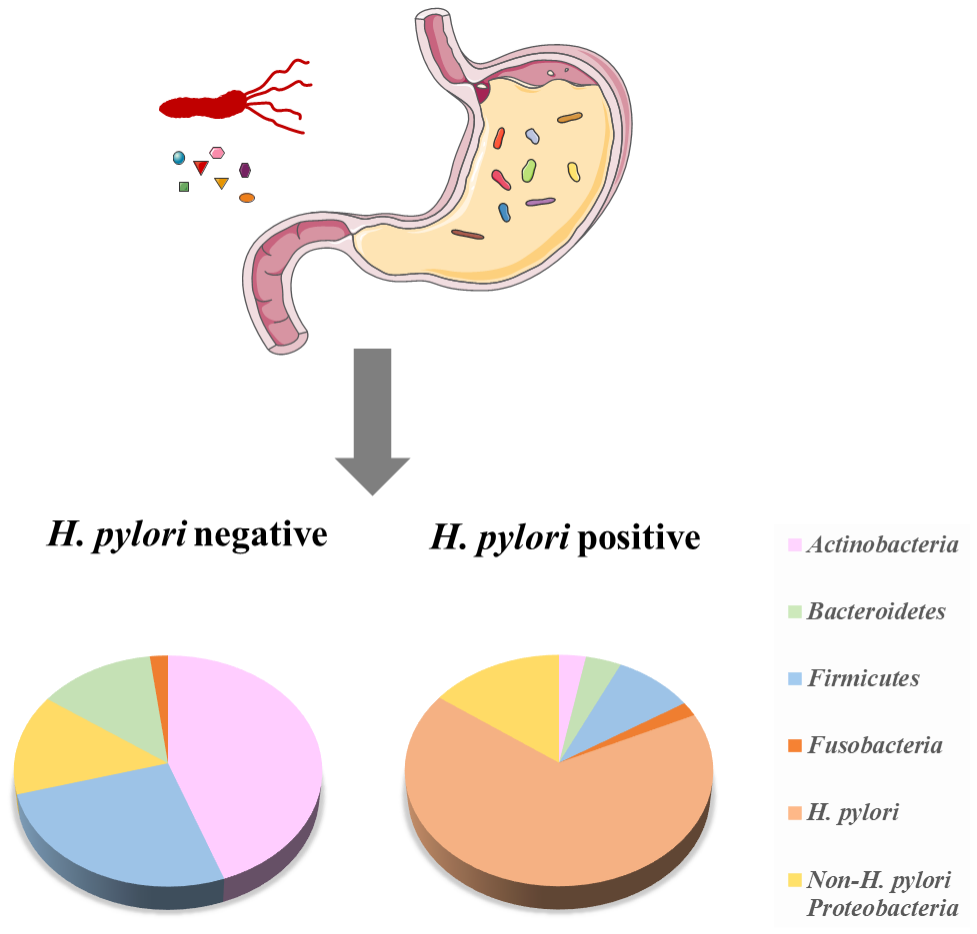
The composition of the human stomach microbiota and the effect of *H. pylori* on the microbiota. The microbiota of *H. pylori* (−) cases has higher diversity, with a higher relative abundance of *Firmicutes* and *Actinobacteria*. *H. pylori* (+) cases have a lower prevalence of *Actinobacteria*, *Bacteroidetes*, and *Firmicutes*, while increase *Proteobacteria* (adopted from Andersson et al., 2008 and Maldonado-Contreras et al., 2011).

In earlier times, Monstein et al. used temperature gradient gel electrophoresis of 16S rRNA amplicons to classify the gastric microbiota into three main phyla (*Proteobacteria*, *Firmicutes*, and *Actinobacteria*; Monstein et al., 2000). As high-throughput sequencing developed, more bacteria were found in the human stomach. G2 PhyloChip (16S rRNA chip) data revealed 44 bacterial phyla in the human stomach, of which 4 phyla dominate: *Actinobacteria*, *Firmicutes*, *Bacteroidetes*, and *Proteobacteria* (Maldonado-Contreras et al., 2011). Based on barcoded 16S pyrosequencing data indicated that the human stomach contains of five phyla: *Actinobacteria*, *Firmicutes*, *Bacteroidetes*, *Proteobacteria*, and *Fusobacteria* (Andersson et al., 2008; Figure 1). In addition,researchers found that it contained the most common genera for each phylum, such as *Streptococcus* (*phylum Firmicutes*), *Neisseria* and *Haemophilus* (*Proteobacteria*), as well as *Prevotella* and *Porphyromonas* (*Bacteroidetes*; Li et al., 2009).

*H. pylori*, a spiral-shaped flagellated bacterium belonging to the Proteobacteria phylum, is considered a constituent of the normal human gastric microbiome (Falush et al., 2003; Linz et al., 2007; Bruno et al., 2018). The remarkable survival capacity of *H. pylori* in the harsh gastric environment can be attributed to its motility and chemotaxis, which facilitate penetration of the mucus layer and colonization of epithelial cells (Amieva and Peek, 2016). *H. pylori* can hydrolyze urea and produce urease to increase the pH of its surrounding environment (Schulz et al., 2018).

Apart from *H. pylori*, many members other than *H. pylori* have been found in the stomach, including *Streptococcus* spp., *Lactobacillus* spp., *Neisseria* spp., *Klebsiella* spp., *Escherichia coli*, *Rothia* sp., *Burkholderia pseudomallei*., *Bacillus* sp., *Morganella morganii*, *Acinetobacter* sp., *Haemophilus* sp., *Veillonella* sp., *Clostridium* sp., *Corynebacterium* sp., *Bacteroides* sp., and *Peptococcus* sp. (Zilberstein et al., 2007; Khosravi et al., 2014; Schulz et al., 2018). Interestingly, some uncultured bacteria correlated with the extremophile *Deinococcus* and members of the enigmatic uncultured bacteria-TM7 group were detected in the stomachs of normal individuals (He et al., 2015; Ye et al., 2016), and another uncultured bacteria-SR1 phylum was also found in the normal stomach (Li et al., 2009).

## Factors affecting gastric microbiota

Various factors are affecting the survival and function of gastric microbiota. The harsh environment in the stomach, which contains antibacterial enzymes, defensins, immunoglobulin, and high gastric acid, was a challenge for gastric microbiota (Zhang et al., 2017). These substances could effectively protect the host’s gastric mucosa from the attack of the microbiota. Low pH in the stomach cavity hinders the growth of the gastric microbiota. The hydrochloric acid secreted by parietal cells can convert pepsinogen into pepsin, an effective enzyme that denatures proteins and inhibits the survival of microbiota (Zhang et al., 2017). Immunoglobulin A (IgA) could prevent bacteria from penetrating the epithelial barrier and potentially maintain the diversity of normal gastrointestinal microbiota (Suzuki et al., 2004). Stomach commercial bacteria, especially S24-7, which belonged to *Bacteroides*, effectively induce the secretion of ILC2-dependent IgA. The secreted IgA is coated with other pathogenic bacteria (such as *H. pylori*) to prevent it from invading the epithelial mucosa, so as to maintain the gastric bacterial homeostasis (Satoh-Takayama et al., 2020). Children’s ILC2 is immature, unable to activate plasma cells to release enough IgA, resulting in *H. pylori* susceptibility (Ohno and Satoh-Takayama, 2020; Satoh-Takayama et al., 2020).Other antimicrobial compounds in gastric epithelial cells produced by the host, such as cathelicidins and C-type lectins, also had selective killing effects on microbiota (Walter and Ley, 2011).

Some external factors also influence the human gastric microbiota, including diet (Cires et al., 2016), antibiotics, proton pump inhibitor (PPI; Nardone and Compare, 2015; Tsuda et al., 2015), geography (Yang et al., 2016), and surgical intervention (Tseng et al., 2016). For instance, in the stomach of healthy cases, the most enriching family was *Prevotellaceae*, followed by *Streptococcaceae*, *Paraprevotellaceae*, and *Fusobacteriaceae*. While among patients who received PPI treatment, *Streptococcaceae* were the prevalent family, followed by *Prevotellaceae*, *Campylobacteraceae*, and *Leptotrichiaceae* (Parsons et al., 2017). Long-term application of PPI increases the intra-gastric pH, which allows the bacteria to reach the growth phase, resulting in increased bacterial load and increased bacterial translocation (Scarpignato et al., 2016), leading to a new bacterial balance, in which oral bacteria are significantly increased, such as *Pepto-streptococcus stomatis*, *Parvimonas micra*, *Slackia exigua*, *Streptococcus anginosus*, and *Dialist pneumonitis* (Bruno et al., 2019). Besides, the diversity of gastric microbiota increased significantly after subtotal gastrectomy. *Helicobacter* and *Ralstonia* were the two most abundant genera in stomachs before surgery, while *Prevotella* and *Streptococcus* were the two most prevalent bacteria after surgery (Tseng et al., 2016). The parietal cells that secrete gastric acid are mainly located in the stomach body and gastric antrum, so the gastric acid secretion is significantly reduced after distal gastrectomy, as well as highly selective vagotomy and bile reflux after surgery, all of which increase the pH value in the stomach and change the composition of bacteria.

## Changes of gastric microbiota in gastric cancer

The dysbiosis of gastric microbiota may be responsible for gastric malignancies. The microbiota changes aggravated gastric environmental disorders and promoted the development of GC (Lofgren et al., 2011; Lertpiriyapong et al., 2014; Guo et al., 2022). The detailed changes in the bacterial community in GC patients were listed as follows (Table 1).

**Table 1:** Changes of gastric microbiota in GC.

Reference	Study participants	Samples	Methods	Significant outcomes
Aviles-Jimenez et al. (2014)	NAG (5), IM (5), GC (5)	Gastric biopsy samples from antrum and corpus	G3 PhyloChip (16S rRNA microarray)	From NAG to IM to GC, decreased bacterial diversity, increased *Lachnospiraceae* and *Lactobacillus coleohominis*, decreased *Porphyromonas, Neisseria* and TM7.
Castaño-Rodríguez et al. (2017)	GC (12), FD (20)	Gastric biopsy samples from antrum, whole blood samples	16S rRNA sequencing V4	Increased richness and phylogenetic diversity but not Shannon’s diversity in GC, enriched *Veilonella*, *Lactococcus*, and *Fusobacteriaceae* (*Leptotrichia* and *Fusobacterium*); enriched short chain fatty acid production pathways in GC.
Chen et al. (2019)	GC (62)	Pairs of matched GC tissues and adjacent non-cancerous tissues	16S rRNA sequencing V4-V5	Increased oral bacteria (*Fusobacterium, Streptococcus* and *Peptostreptococcus*) in tumor tissues, increased lactic acid-producing bacteria (*Lactobacillus brevis* and *Lactococcus lactis*) in adjacent non-tumor tissues.
Png et al. (2022)	EC (4), IM (22), SG (17)	Gastric biopsy samples from antrum	16S rRNA sequencing V3-V4	Increased the abundances of Proteobacteria (in particular Proteus genus) in EC. Decreased the abundances of Bacteroidetes (in particular S24-7 family)
Coker et al. (2018)	GC (20), IM (17), AG (23), SG (21) Validate: C (19), AG (51), SG (56)	Gastric biopsy samples from antrum, body and fundus	16S rRNA sequencing V4	Enriched *Dialister pneumosintes, Parvimonas micra, Peptostreptococcus stomatis, Slackia exigua* and *Streptococcus anginosus* in GC.
Dai et al. (2021)	GC (37)	Pairs of matched GC tissues and adjacent non-cancerous tissues	16S rRNA sequencing V3-V4	Increased the abundances of Lactobacillus, Prevotella, Streptococcus, Acinetobacter, Sphingomonas, Bacteroides, Comamonas, Fusobacterium, Empedobacter, and Faecalibacterium in the tumor tissues.
Dicksved et al. (2009)	GC (10), FD (5)	Gastric biopsy samples from antrum and corpus	T-RFLP,16S rRNA sequencing V3	Increased *Streptococcus, Lactobacillus, Veillonella* and *Prevotella*, decreased *H. pylori* in GC.
Eun et al. (2014)	GC (11), IM (10), CG (10)	Gastric biopsy samples	16S rRNA sequencing V5	Increased the diversity of gastric microbiota, increased *Streptococcaceae* and *Bacilli* at the class level, decreased *Helicobacter* at the familiy level in GC.
Ferreira et al. (2018)	CG (81), GC (54)	Gastric biopsy samples	16S rRNA sequencing V5-V6	Decreased microbial diversity, decreased *Helicobacter* abundance and increased other bacterial genera (include intestinal commensals) in GC.
Hsieh et al. (2018)	CG (9), IM (7), GC (11)	Gastric biopsy samples	16S rRNA sequencing V3-V4	Similar abundance of *Burkholderia, Enterobacter,* and *Leclercia* in cancer and non-cancer groups. Increased the abundance of *Fusobacterium*, *Lactobacillus* and *Clostridium*; decreased the abundance of *H. pylori* in GC.
Hu et al. (2018)	SG (5), GC (6)	Gastric wash samples	shotgun metagenomic sequencing	Decreased species richness in GC group, especially *Sphingobium yanoikuyae*, increased 13 bacterial taxa and decreased 31 taxa in GC; genera *Aggregatibacter, Alloprevotella and Neisseria* in GC were different from SG. Enriched L-arginine and lipopolysaccharide production pathways in GC.
Jo et al. (2016)	*H. pylori* (−) control (13)*, H. pylori* (+) control (16), *H. pylori* (−) GC (19), *H. pylori* (+) GC (15)	Gastric biopsy samples from antrum and corpus, blood samples	16S rRNA sequencing V1-V3	Increased the proportion of *Actinobacteria* in GC groups. *Stenotrophomonas* genus (*Stenotrophomonas maltophilia*) was the most abundant in *H. pylori* (−) GC group, while *Helicobacter* genus was the most abundant in *H. pylori* (+) GC group.
Li et al. (2017)	CG (9), IM (9), GC (7), *H. pylori* (−) control (8)	Gastric biopsy samples from antrum and corpus	16S rRNA sequencing V3-V4	Decreased microbial diversity, enriched the abundance of 13 high OTUs (e.g.*Flavobacterium, Klebsiella, Serratia marcescens*, *Stenotrophomnonas*, *Achromobacter* and *Pseudomonas*) in GC group.
Ling et al. (2019)	GC (64)	Normal, peritumoral and tumoral tissues	16S rRNA sequencing V3-V4	Composition, diversity and function of gastric microbiota changed more obvious in tumoral tissues than in normal and peritumoral tissues.
Liu X. et al. (2019)	GC (276)	Normal, peritumoral and tumoral tissues	16S rRNA sequencing V3-V4	Decreased bacterial richness in tumoral and peritumoral tissues, decreased the abundance of *H. pylori*, *Bacteroides uniformis* and *Prevotella copri*, increased the abundance of *Propionibacterium acnes, Streptococcus anginosus* and *Prevotella melaninogenica* in tumoral tissues.
Gunathilake et al. (2019)	GC (268), non-cancer-bearing controls (288)	Gastric biopsy samples	16S rRNA sequencing V3-V4	Increased the abundances of *H. pylori* and *Prevotella copri*, *Propionibacterium acnes*, decreased the abundances of *Lactococcus lactis* in GC.
Park et al. (2019)	GC (55), IM (19), CG (62)	Gastric biopsy samples from antrum	16S rRNA sequencing V3-V4	Increased the abundances of *Moraxellaceae, Pseudomonadaceae, Streptococcaceae* and *Xanthomonadaceae* in *H. pylori* (−) GC compared to *H. pylori* (−) CG and *H. pylori* (−) IM groups. Decreased the abundances of *Cyanobacteria* and *Rhizobiales* in *H. pylori* (−) GC.
Seo et al. (2014)	GC (16)	Tumor and non-tumor biopsy samples	16S rRNA sequencing	Increased *Prevotella spp.* and *Clostridium* spp. in tumor tissue, decreased *Corynebacterium* spp., *Propionibacterium* spp. and *Staphylococcus* spp. at the genus level, and decreased *H. pylori* at the species level in tumor tissue.
Shao et al. (2019)	GC (36)	Gastric tumor tissues, non-malignant tissues	16S rRNA sequencing V4	Increased the abundances of *Haemophilus, Neisseria, Prevotella*, *Streptococcus* and *Veillonella*, decreased the abundance of *Helicobacter* genus in tumor tissues. Increased the abundance of *Helicobacter* in non-tumor tissues.
Sohn et al. (2017)	*H. pylori* (−) control (2), *H. pylori* (+) control (3), *H. pylori* (−) GC (2), *H. pylori* (+) GC (5)	Gastric biopsy samples from antrum and body	16S rRNA sequencing V1-V3	Increased the number of non- *H. pylori* urease-producing bacteria and non- *H. pylori* nitrosating or nitroreducing bacteria (e.g., *S. pseudopneumoniae, S. parasanguinis*, and *S. oralis*) in *H. pylori* (−) GC groups.
Sun et al. (2022)	*H. pylori* (−) SG (56), *H. pylori* (−) AG (9), *H. pylori* (−) IM (27), *H. pylori* (−) Dys (29), *H. pylori* (−) GC (13).	Gastric mucosal biopsy samples, and Gastric juice	16S rRNA sequencing V3-V4	Increased the abundances of Burkholderiaceae, decreased the abundance of Streptococcaceae and Prevotellaceae
Tseng et al. (2016)	GC (6)	Gastric cancerous tissues, adjacent normal tissues	16S rRNA sequencing V1-V3	Increased *Ralstonia* and *Helicobacter* in cancerous tissues before surgery; increased *Streptococcus* and *Prevotella* in cancerous tissues after surgery, increased the diversity of gastric microbiota after surgery.
Wang et al. (2016)	GC (103), CG (212)	Gastric biopsy samples	16S rRNA sequencing V1-V3	Increased the quantity and diversity of bacteria, enriched bacteria with potential cancer-promoting activities in GC.
Wang L. et al. (2020)	EC (30),AC (30),CG (60)	Gastric mucosal biopsy, adjacent normal tissues	16S rRNA sequencing V3-V4	Increased the levels of *Ochrobactrum*,*Lactobacillus,Propionibac,serratiaterium* et al.*in EC.*
Wang et al. (2020b)	CG (21), IM (27), IN (25), GC (29), non-cancer-bearing controls (30)	Gastric mucosal biopsy	16S rRNA sequencing V4	Decreased the diversity and abundances of phyla *Nitrospirae*, *Chloroflexi*, *Armatimonadetes*, *Elusimicrobia*, *Verrucomicrobia*, *Planctomycetes* and WS3 from CG, IM, IN to GC. Enriched *Bacteriodes, Actinobacteria, Fusobacteria, Firmicutes,* TM7, and SR1 in the IN and GC group.
Wu et al. (2020)	GC (18), SG (32)	Paired tumor and paracancerous mucosa samples	16S rRNA sequencing	Increased the levels of *Lactobacillus spp. Dialister spp., Rhodococcus spp., Helicobacter spp., Sediminibacterium* spp. and *Rudaea* spp. in GC, decreased species *Fusobacterium spp., Actinomyces spp., Brevundimonas spp., Leptotrichia spp., Haemophilus spp., Alloprevotella spp., Campylobacter spp., Arthrobacter spp., Neisseria spp., Bradyrhizobium spp., Phyllobacterium spp., Prevotella spp., Porphyromonas spp., Veillonella* spp. and *Rothia* spp., etc. in GC*.*
Yu et al. (2017b)	GC (77)	Gastric tumor tissues, paired non-malignant tissues	16S rRNA sequencing	Decreased *H. pylori* and increased *Bacteroidetes* abundance in lower tumor grade. Increased *H. pylori* abundance and decreased alpha diversity in advanced tumor grade. Class *Epsilonproteobacteria,* order *Campylobacterales*, family *Helicobacteraceae*, and genus *Helicobacter*, were also related to tumor grade.
Yu et al. (2017a)	GC (160)	Gastric tumor tissues, paired non-malignant tissues	16S rRNA sequencing V3-V4	Dominated *Proteobacteria*, followed by *Bacteroidetes* in Chinese tumor samples or *Firmicutes* in Mexican tumor samples. Dominated *H. pylori* in both Chinese and Mexican tumor tissues, but *H. pylori* abundance is lower than that of matched non-malignant tissues.

GC, gastric cancer; AC, advanced gastric cancer; EC, early gastric cancer; IM, intestinal metaplasia; AG, atrophic gastritis; SG, superfcial gastritis; NAG, non-atrophic gastritis; CG, chronic gastritis; FD, functional dyspepsia; AH, atypical hyperplasia; IN, intraepithelial neoplasia; Dys, dysplasia.

GC patients were more likely to suffer from acid deficiency, which may affect the colonization of microorganisms. In the early stages of GC, the relative abundance of *H. pylori*, *Propionibacterium acnes*, and *Prevotella copri* in the stomach was higher than that of non-cancer-bearing people (Gunathilake et al., 2019). However, Wang LL et al. found that unlike advanced gastric cancer, no significant biodiversity alteration was found in the early stage of gastric cancer (Wang L. et al., 2020). As cancer develops, the prevalence of *H. pylori* in the stomach may gradually decrease, and the overall microbial population in the stomach may also change. Dicksved et al. found a decreased abundance of *H. pylori* in the stomach of GC patients and a predominance of different species of the gastric microbial population, which include the genera *Lactobacillus*, *Streptococcus, Prevotella*, and *Veillonella* (Dicksved et al., 2009). Coker et al. found five taxa (*Dialister pneumosintes*, *Parvimonas micra*, *Peptostreptococcus stomatis*, *Slackia exigua*, and *Streptococcus anginosus*) as the core of the GC microbiota network (Coker et al., 2018). These studies revealed the difference in gastric microbiota profiles between GC patients and non-cancer-bearing people.

The gastric microbiota also showed different changes in patients with different histological stages from gastritis to GC. Ferreira et al. confirmed that the dysbiosis of gastric microbiota with potential genotoxicity existed in the GC patients, which differed from that of patients with chronic gastritis (CG; Ferreira et al., 2018). From non-atrophic gastritis and intestinal metaplasia (IM) to GC, the abundance of *Neisseria*, *Porphyromonas*, *Streptococcus sinensis*, and TM7 group showed a decreasing trend. In contrast the abundance of *Lachnospiraceae* and *Lactobacillus coleohominis* displayed an increasing trend (Aviles-Jimenez et al., 2014). In another study, compared with CG and IM groups, the abundance of *Streptococcaceae* and *Bacilli* increased at the class level, and the abundance of *Helicobacter* decreased at the family level in the stomach of the GC patients (Eun et al., 2014). In *H. pylori*-negative patients from atrophic gastritis (AG) to dysplasia (Dys) precancerous stage, the abundance of *Burkholderiaceae* continued to increase, while the abundance of *Streptococcaceae* and *Prevotellaceae* continued to decrease (Sun et al., 2022). Interestingly, some oral bacteria, genera *Aggregatibacter*, *Alloprevotella*, and *Neisseria* were abundant in GC patients compared with the superficial gastritis (SG) group. The relative abundance of these bacteria was completely separated between the two groups. This discovery suggested we can distinguish GC from SG patients based on any of the three genera detected in GC (Hu et al., 2018).

Previous studies have primarily focused on the abundance and diversity of the gastric microbiota in diverse patient cohorts. However, uncertain host factors may exert significant influence on research outcomes, thereby confounding interpretation of the results. To minimize the impact of these confounding factors, some investigators have adopted a paired design approach, wherein the profiles of the gastric microbiota in paired tumor tissues and non-malignant tissues from the same GC patient are comparatively analyzed in greater detail. Seo et al. detected 350 bacterial species from paired cancer and non-cancer biopsies among 16 GC patients by 16S rRNA gene sequencing. Compared with non-cancer tissue, the populations of *Prevotella* spp. and *Clostridium* spp. were increased, while *H. pylori*, *Propionibacterium* spp., *Staphylococcus* spp., and *Corynebacterium* spp. were decreased in cancer biopsies (Seo et al., 2014; Dai et al., 2021). In a study of carcinoma and adjacent tissues from 276 GC patients, genera *Halomonas*, *Shewanella*, and *Helicobacter* were enriched in the tumor-adjacent tissues, while *Corynebacterium*, *Fusobacterium*, *Selenomonas*, *Propionibacterium*, and *Streptococcus* were enriched in the carcinoma tissues. Similar to the previous reports, the community of *H. pylori* was also *significantly* reduced in the tumoral sites (Liu X. et al., 2019). The significant reduction of *H. pylori* may be due to the changes in the gastric environment of GC patients.

There are significant differences in the composition of gastric microbiota in GC patients based on race and region. A Portuguese cohort study showed an increased abundance of *Achromobacter* in GC patients compared to gastritis patients, while *Achromobacter* was completely absent in the validation cohort of Chinese GC subjects (Ferreira et al., 2018). Yu et al. analyzed bacterial abundance and diversity in GC tissues from 80 Chinese and 80 Mexican patients. Similar to the gastric microbiota profiles in non-cancer tissues, microbiota in cancer tissues of Mexican and Chinese patients were also composed mainly of *Proteobacteria*, followed by *Firmicutes* in Mexican cancer tissues or *Bacteroides* in Chinese cancer tissues. Mexican samples showed an increased relative abundance of *Clostridium* in cancer tissues but no difference in the alpha diversity, while cancer samples from Chinese patients presented substantial differences in alpha diversity and the abundance of several genera has increased, such as *Treponema*, *Helicobacter*, *Selenomonas*, *Fusobacterium*, *Streptococcus*, *Pseudomonas* (Yu et al., 2017a). These studies indicated that geographical and ethnic factors could influence the composition of stomach microbes.

Notably, a robust correlation existed between stomach microbes and the epidemiology of GC. Compared with cases without a family history of upper gastrointestinal cancer, cases with a family history of upper gastrointestinal cancer have lower alpha diversity and a higher abundance of *H. pylori* (Yu et al., 2017b). Among populations with similar *H. pylori* prevalence, the gastric microbiota composition significantly differed in populations from two towns with different GC risks in Colombia. *Leptotrichia wadei* and *Veillonella* sp. were considerably abundant in populations from Túquerres, a town with high GC risk, while *Staphylococcus* sp. were strikingly abundant in populations from Tumaco, a town with low GC risk (Yang et al., 2016). These findings demonstrated that the characteristics of the gastric microbiota in GC patients were associated with both familial history of gastrointestinal tumors and diverse environmental conditions.

## *Helicobacter pylori* and gastric cancer

The first animal experiment on the pathogenicity of *H. pylori* was performed by the Mongolian gerbil model. It revealed that *H. pylori* induced a continuous development from superficial gastritis to pre-malignant lesions (Hirayama et al., 1996). Compared with age-matched uninfected mice, mice infected with *H. pylori* had more severe inflammation, acid atrophy, hyperplasia, epithelial defects, and dysplasia (Lee et al., 2008). Likewise, compared with the control gerbils, low differentiated adenocarcinoma and carcinoid were discovered in the gerbils inoculated with *H. pylori* (Hirayama et al., 1999).

*H. pylori* infection is one of the main causes of gastric cancer and can increase the risk of gastric cancer by 2.2–21 times (Uemura et al., 2001; Suerbaum and Michetti, 2002). *H. pylori* infection could induce chronic inflammation in the stomach, which was accompanied by genetic alterations and DNA damage in gastric epithelial cells. *H. pylori* infection has been found to trigger ubiquitination and proteasomal degradation of p53, a critical regulator of genome stability, thereby impairing the repair of genome damage. *H. pylori* infection reduces the expression of the transcription factor USF1, which can stabilize the function of P53, and thereby increasing viability of gastric epithelial cells with persistent DNA damage and promoting gastric carcinogenesis (Costa et al., 2020). *H. pylori* could also downregulate the expression of genes associated with tumor suppression by inducing abnormal DNA methylation (Servetas et al., 2016; Choi et al., 2020). Abnormal DNA methylation in the gene promoter region leads to the inactivation of tumor suppressor and other cancer-related genes in cancer cells, which is the most clear epigenetic marker in gastric cancer (Qu et al., 2013). Chan AO et al. observed that *H. pylori* infection caused E-cadherin methylation to be more frequent in the gastric mucosa compared to cases without *H. pylori* infection (Chan et al., 2003). Maekita et al. found that *H. pylori* infection can effectively induce CpG islands methylation to varying degrees (Maekita et al., 2006). *H. pylori* infection also delays gastric epithelial cell apoptosis (Sáenz and Mills, 2020; Imai et al., 2021). *H. pylori* infection induced an increase in cellular spermine oxidase (SMOX), and phosphorylated EGFR (pEGFR), resulting in the generation of a subpopulation of gastric epithelial cells with high levels of DNA damage and resistance to apoptosis (Chaturvedi et al., 2011, 2014).

Among possible explanations of GC caused by *H. pylori*, the two most widely accepted virulence factors were Cytotoxin-associated gene A (*CagA*) and Vacuolating cytotoxin A (*VacA*; Amieva and Peek, 2016), which have been linked to the carcinogenic potential of this bacterium. The *CagA* gene is the most important pathogenic factor of *H. pylori*. Compared with the strains without *CagA*, strains containing *CagA* increase the risk of gastric cancer by 1.64-fold overall (Censini et al., 1996; Huang et al., 2003). *Cag* (+) *H. pylori* induced TP53 gene mutation and aberrant expression of activation-induced cytidine deaminase, which may be responsible for the accumulation of mutation in gastric carcinogenesis (Matsumoto et al., 2007; Yong et al., 2015). *CagA* (+) *H. pylori* infection caused the activation of multiple oncogenic pathways, including ERK/MAPK, PI3K/AKT, NF-kB, Wnt/β-catenin, Ras, Hippo, and STAT3 (Udhayakumar et al., 2007; Salama et al., 2013; Yong et al., 2015; Imai et al., 2021). *CagA* also disturbed the host’sepithelial cells, precursor cells and stem cells (Bessède et al., 2014; Wroblewski et al., 2015). Another virulence factor, *VacA*, was involved in regulating immune responses and autophagy. *VacA* regulated host cell metabolism by inhibiting mTORC1 and promoted gastric epithelial cell apoptosis by interfering with the function of mitochondria (Kim et al., 2018). In addition, *VacA* promotes Treg differentiation by inducing dendritic cell expression and releasing some anti-inflammatory cytokines, such as IL-18 and IL-10, thus suppressing anti-tumor immunity (Kao et al., 2010; Oertli et al., 2012). Prolonged exposure to *VacA* can interrupt autophagy, which is manifested by the accumulation of P62. Autophagy is an important protective mechanism of the stomach against *H. pylori* infection. The interruption of this mechanism will cause cell death, inflammation and genetic instability, forming a microenvironment prone to cancer (Raju et al., 2012).

Other pathogenic mechanisms of *H. pylori* have also been widely reported. Some adhesins, such as sialic acid-binding adhesin (SabA), blood-antigen binding protein A (BabA) and neutrophil-activating protein (NAP), attached to host cell receptors and increased risk of peptic ulcer and GC (Kao et al., 2016). Targosz et al. proved that *H. pylori* up-regulated the expression of cyclooxygenase-2 (COX-2) mRNA in gastric epithelial cells, which was known to be a carcinogenesis-related rate-limiting enzyme (Targosz et al., 2012; Shao et al., 2014). The accumulation of activated β-catenin in the nucleus of gastric epithelial cells induced by *H. pylori* was closely connected with tumor invasion (Cheng et al., 2004), indicating the aberrant activation of β-catenin may be a key member in regulating pre-malignant epithelial responses to *H. pylori.* In addition, *H. pylori* infection induced the expression of hepatoma-derived growth factor (HDGF), which stimulated the differentiation of human mesenchymal stem cells into myofibroblast-like cells and further promoted the survival and invasion of human GC cells (Liu et al., 2018).

## Other gastric microbiota and gastric cancer

Under conditions of absent acidity (such as AG and IM), some non-*H. pylori* bacteria produce active oxygen or nitrogen to regulate inflammatory reactions, and the gastric micro-ecosystem became more complex (Sheh and Fox, 2013). The abundance of some genera showed a consistent increase in GC patients, including *Staphylococcus* (Jo et al., 2016; Castaño-Rodríguez et al., 2017; Shen et al., 2022), *Lactobacillus* (Castaño-Rodríguez et al., 2017; Coker et al., 2018; Ferreira et al., 2018), *Clostridium* (Castaño-Rodríguez et al., 2017; Ferreira et al., 2018), *Fusobacterium* (Castaño-Rodríguez et al., 2017; Coker et al., 2018), *Streptococcus* (Dicksved et al., 2009; Castaño-Rodríguez et al., 2017; Coker et al., 2018), *Bifidobacterium*, and *Lactococcus* (Castaño-Rodríguez et al., 2017). These bacteria had different effects on stomach pathogenesis. A prospective study concluded that the characteristics of gastric microbiota in non-tumor patients could accurately classify patients who may develop EC. They identified a constellation of six bacterial taxonomic markers, including the *Moryella genus*, *Vibro genus*, *Comamonadaceae*, *Paludibacter*, *Agrobacterium*, and *Clostridiales* (Png et al., 2022).

Some non-*H. pylori* bacteria promote the inflammatory response to accelerate the progression of GC, such as *Lactobacillus murinus*, *Clostridium,* and *Streptococcus salivarius* (Lertpiriyapong et al., 2014). It has been proved that the overgrowth of *Propionibacterium acnes* may contribute to lymphocytic gastritis through *in vitro* cell experiments. Lymphocytic gastritis caused by *Propionibacterium acnes* produces pro-inflammatory cytokine IL-15, which is a potential trigger for GC (Montalban-Arques et al., 2016). *Prevotella* had the classification ability to distinguish GC patients from non-cancer-bearing people with an area under the curve of 0.76 (Wu et al., 2018). The pathogenicity of *Prevotella copri* has been proven to produce redox proteins in the human body (Hofer, 2014; Wu et al., 2018). Besides, the Insulin-Gastrin (INS-GAS) transgenic mice colonized *Lactobacillus murinus*, *Clostridium* and *Bacteroides* developed gastrointestinal intraepithelial neoplasia, which strongly related to the upregulation of pro-inflammatory and oncogenic genes (Lertpiriyapong et al., 2014). The microflora of non-*H. pylori* in the stomach also influenced the severity of *H. pylori*-induced gastric cancer. Shen et al. found that *Streptococcus salivarius* coinfection with *H. pylori* induced significantly higher gastric pathology than in *H. pylori*-monoinfected mice. In contrast, *Staphylococcus* epidermidis coinfection caused significantly lower *H. pylori*-induced pro-inflammatory cytokine responses than in *H. pylori*-monoinfected mice (Shen et al., 2022).

Some non-*H. pylori* bacteria could disturb the function of immune cells in the tumor microenvironment to promote GC. Previous studies found a positive correlation between *Stenotrophomonas* in GC tissues and plasmacytoid dendritic cells that have the function of suppressing immune effector cells (Huang et al., 2014; Ling et al., 2019). Similarly, in GC microhabitats, *Selenomonas* was positively associated with regulatory T cells with immunosuppressive effects (Ahmetlić et al., 2019; Ling et al., 2019). These studies suggest *Selenomonas* and *Stenotrophomonas* may promote cancer cells to evade surveillance by the immune system. Besides, it was reported that *Fusobacterium nucleatum* disturbed the phenotypes and functions of immune cells such as neutrophils, T cells, NK cells, dendritic cells and macrophages, forming an immunosuppressive microenvironment conducive to cancer growth (Wu et al., 2019). The increase of *Fusobacterium* in *H. pylori* (−) GC patients may be related to this mechanism (Hsieh et al., 2018). Li Q et al. found that excess *Propionibacterium acnes* promotes gastric cancer progression by promoting M2 polarization of macrophages through TLR4/PI3K/Akt signaling (Li et al., 2021).

Non-*H. pylori* bacteria produced metabolites that may promote the occurrence of GC. Higher levels of non-*H. pylori* nitrosated or nitrate-reducing bacteria (NB) and non-*H. pylori* urease-producing bacteria (UB) were found in *H. pylori* (−) GC patients (Jo et al., 2016; Sohn et al., 2017). It is well known that N-nitroso compounds (NOCs) are potent carcinogens (Hernández-Ramírez et al., 2009; Jo et al., 2016). NOCs formed from nitrite and secondary amines and were observed in some nitrate-reducing gastric bacteria, including *Clostridium*, *Veillonella*, *Haemophilus*, *Staphylococcus*, *Streptococcus*, and *Neisseria* (Ayanaba and Alexander, 1973; Hu et al., 2012; Jo et al., 2016). Similarly, urease is the main trigger of innate immune response produced by various non-*H. pylori* such as *Lactococcus*, *Clostridium*, *Haemophilus*, and *Actinomyces* (Sohn et al., 2017). These nitrate-reducing and urease-producing bacteria may be involved in the pathological mechanism of stomach disorders. However, the detailed pathogenic mechanism remains to be further confirmed.

Not all microbes in the stomach are harmful, and some studies have found the presence of bacteria in the stomach that can inhibit the progression of gastric cancer. For example, Kim SY et al. found that *Lactococcus lactis ssp. lactis* can affect the expression of p53 and p21 to induce cell cycle arrest and apoptosis to inhibit the proliferation of gastric cancer cells (Kim et al., 2004, 2009). In addition, Hwang CH et al. studied that Heat-Killed *Lactobacillus* can induce the expression of pro-apoptotic genes and inhibit the proliferation of gastric cancer cell line AGS *in vitro*. However it still needs to be verified by *in vivo* experiments (Hwang et al., 2022).

## Interaction between *Helicobacter pylori* and other gastric microbiota

*H. pylori* infection altered the composition of the human stomach microbiome (Figure 1). Compared with the gastric microbiota of healthy cases, *H. pylori*-infection individuals have a lower diversity, with a lower abundance of *Actinobacteria*, *Firmicutes* and *Bacteroidetes*, while increased *Proteobacteria* (Andersson et al., 2008; Maldonado-Contreras et al., 2011).In *H. pylori* (+) GC patients, the proportion of the *Streptococcus mitis* group (such as *S. oralis*, *S. infantis*, *S. mitis*, *S. tigurinus,* and *S. pseudopneumoniae*) was significantly lower than that of the *H. pylori* (−) GC group (Sohn et al., 2017). These reduced bacteria may be related to the unfavorable conditions caused by *H. pylori* infection. The tendency of co-occurrence/co-competition among gastric microbiota has been further investigated. Das et al. found that in *H. pylori* (+) patients, *H. pylori* showed a negative association (inhibit other bacteria) with some gastric members, such as *Acidovorax*, *Aeromonas*, *Bacillus*, *Bradyrhizobium*, *Halomonas*, *Cloacibacterium*, *Meiothermus*, *Methylobacterium*, and *Ralstonia*, while the interactions of these non-*H. pylori* members were positively correlated (das et al., 2017).

The possible influence of *H. pylori* on the structure and function of the gastric microbes has been demonstrated by animal experiments. The addition of restricted microbiota (*Clostridium* ASF356, *Bacteroides* ASF519, *Lactobacillus* ASF361) in the stomach of INS-GAS mice was sufficient to promote gastric mucosal lesions such as moderate inflammation, gland atrophy, epithelial defects, dysplasia, but no gastrointestinal intraepithelial neoplasia. However, mice co-infected with these restricted bacteria and *H. pylori* developed higher-grade glandular abnormalities, and 69% of mice with dysplasia were identified as gastrointestinal intraepithelial neoplasia compared with mice infected only with restricted bacteria (Lertpiriyapong et al., 2014). It indicated that *H. pylori* synergistically accelerated the onset and progression of gastrointestinal intraepithelial neoplasia in mice infected with restricted bacteria. One year after inoculation with *H. pylori*, the numbers of *Atopobium* cluster increased and *Bifidobacterium* spp., *C. coccoides* group, and *C. leptum* subgroup decreased in *H. pylori* (−) gerbils compared to the uninfected gerbils. Besides, *Prevotella spp.* and *Eubacterium cylindroides* group were absent in *H. pylori* (+) gerbils (Osaki et al., 2012). These results suggested that infection with *H. pylori* for a long time may disturb the composition of the gastric microbiota in mice (Osaki et al., 2012). Similarly, the localization and levels of *Bifidobacterium spp.*, *Bacteroides spp.*, *Enterococcus spp.*, *Staphylococcus aureus* and aerobes were modified and caused more severe gastritis in Mongolian gerbils after *H. pylori* infection. Prolonged colonization of *H. pylori* made the stomach environment unsuitable for the reproduction of lactobacilli, while *Bacteroides*, *Bifidobacteria*, *S. aureus* and *Enterococci* could better adapt to the stomach environment (Yin et al., 2011).

*H. pylori* eradication studies have also demonstrated the relationship between the growth of non-*H. pylori* and *H. pylori.* An inverse correlation was observed between the bacterial diversity and relative abundance of *H. pylori* in GC patients. Compared with non-GC patients with similar levels of *H. pylori*, GC patients showed lower bacterial diversity. After the eradication of *H. pylori*, the diversity of the gastric microbes was increased, and the microbiota abundance was restored to be similar to that of cases without *H. pylori* infection (Li et al., 2017). Likewise, *H. pylori* in the stomach inhibited the colonization of *Enterobacteria*, *Clostridium leptum* and *Lactobacillus*. After eradicaing of *H. pylori*, the bacteria in the patient’s stomach increased significantly (Li et al., 2016). In another study, in the *H. pylori* (+) patients, the total number of non- *H. pylori*- NB decreased in the eradicated gastric biopsies and increased in the non-eradicated or failed to eradicate samples (Jo et al., 2016). These studies indirectly confirmed the role of *H. pylori* infection in disturbing gastric microbiota composition.

To sum up, the mechanism of gastric microbiota causing GC is a multi-factor and multi-step process. *H. pylori* is the main trigger of histopathological changes in GC, and its interactions with non- *H. pylori* are jointly involved in the development of GC. *H. pylori* may be more important in the early stages of GC. But the state of achlorhydric induced by *H. pylori* can disturb gastric microbiota, which may play a key role in the later stages of GC (Dias-Jácome et al., 2016).

## Regulation of gastric microbiota

### Medication

Among the drug interventions to reduce the risk of GC, one of the most studied approaches is *H. pylori* eradication therapy. All patients who test positive for *H. pylori* should be offered eradication therapy. The internationally recommended treatment is the combination of PPI, 2–3 antibiotics, and bismuth, and it should be taken in strictly accordance with the course of treatment. Antibiotic abuse and irregular medication during the treatment of *H. pylori* have made *H. pylori* resistant to clarithromycin, levofloxacin, metronidazole and other drugs. The combination of drugs and the course of treatment vary according to different populations. The main recommended first-line treatment options were Bismuth quadruple therapy, Concomitant therapy, Sequential therapy, Levofloxacin triple therapy and so on (Chey et al., 2017). After 8 weeks of eradication treatment for CG or IM patients, *H. pylori* was significantly reduced, and the diversity of the microbiota increased (increased 31 operational taxonomic units; Li et al., 2017). It indicated that eradication treatment restored the diversity of gastric microbiota. Some studies have shown that eradicating *H. pylori* effectively alleviated stomach pathology (Bae et al., 2018; Sakitani et al., 2018). Eradication treatment also reduced the incidence of GC in healthy cases and patients with gastric neoplasia, reducing GC-related mortality (Choi et al., 2018; Doorakkers et al., 2020; Ford et al., 2020). However, there is evidence that *H. pylori*-eradicated patients were associated with an increased risk of GC (Cheung et al., 2019). Therefore, the effect of *H. pylori* eradication on the incidence of GC still needs to be clarified.

### Probiotics and dietary

Probiotics and dietary fiber regulate the gastrointestinal microbiota and immune response; supplementing them was considered a preventive intervention (Zhang et al., 2013; Hill et al., 2014). It was reported that probiotics increased the eradication rate of *H. pylori* and reduced the incidence of side effects of antibacterial treatment (especially diarrhea; de Bortoli et al., 2007; Ojetti et al., 2012; Wang et al., 2013; Keikha and Karbalaei, 2021). It is worth noting that probiotics are not effective *per se* and can only be used as adjunctive therapy for clinical improvement (Wang et al., 2013). Probiotics also improve anticancer properties by producing lactic acid and other organic acids to inhibit the growth of microorganisms that produce mutagens and carcinogens (Dugas et al., 1999). *Lactobacillus* spp. is one of the best-known probiotics and their anti-*H. pylori* properties have been proven (Bahmanyar and Ye, 2006).Apart from probiotics, prebiotics and dietary fiber also synergistically affected *H. pylori* eradication therapy and were strongly associated with a lower risk of GC (Zhang et al., 2013; Shafaghi et al., 2016). Wheat bran acted as a nitrite scavenger, potentially offsetting the carcinogenic effects of NOCs produced by nitrate-reducing bacteria (Møller et al., 1988). The inhibitory effect of garlic on the growth of *H. pylori* was observed (Jonkers et al., 1999). The antibacterial properties of garlic may be attributed to allicin. Allicin has been confirmed to have a direct antibacterial effect on the growth of *H. pylori in vitro* (Cañizares et al., 2004).

Studies have reported synergistic relationships between multiple dietary components, such as vegetables, fruits, pickles, and soy products, in the development of GC (Bahmanyar and Ye, 2006).Fresh vegetables contain a various of antioxidants that acted as protectants, potentially ameliorating the effects of microbial dysbiosis (Epplein et al., 2008). Broccoli sprouts were rich in sulforaphane, the sulforaphane which had a strong bactericidal effect on *H. pylori* (Yanaka et al., 2009). Dietary patterns of high vegetables and seafood were associated with lower gastric dysbiosis index and lower the risk of GC in males. Sheu et al. pointed out that yogurt containing *lactobacilli* and *bifidobacteria* can improve the cure rate of *H. pylori* infection (Sheu et al., 2002).And the high-dairy dietary pattern was associated with a lower gastric dysbiosis index to reduce GC risk in females (Gunathilake et al., 2021). The intake of red meat and processed meat is related to the increased risk of gastric cancer, especially in H. pylori (+) subjects (González et al., 2006; Huang et al., 2021). Salt induces gastritis by directly damaging the gastric mucosa and increasing the rate of mitosis, and excess salt intake enhances *H. pylori* colonization. Therefore, long-term excessive salt intake will increase the risk of gastric cancer (Yamaguchi and Kakizoe, 2001; Peleteiro et al., 2011; D’Elia et al., 2012; Smyth et al., 2020). Therefore, the change of dietary structure, including reducing the intake of salt and red meat, and increasing the intake of vegetables and fruits, is a possible strategy to prevent gastric cancer.

### Transplantation of rumen microbes

In recent years, fecal bacteria transplantation has been a focus to restore the healthy microbiota of the recipient. This effective strategy has been demonstrated in the treatment of various diseases, such as recurrent *Clostridium difficile* infection (Garza-González et al., 2019), inflammatory bowel disease (Colman and Rubin, 2014) and cancers (McQuade et al., 2020; Baruch et al., 2021). Liu et al. investigated the beneficial effects of rumen fluid transplantation on rumen morphology and function in a sheep model of rumen acidosis. Rumen fluid transplantation accelerated the rapid reconstruction of bacterial homeostasis in the rumen from an obvious acidosis state to a healthy level (similar to that of the donor). Furthermore, it reduced the damage of rumen epithelial cells caused by acute rumen acidosis (Liu J. et al., 2019). The results indicated that rumen microbiota transplantation is a promising strategy for reconstructing bacterial homeostasis. However, rumen transplantation is only an early attempt at the animal level, and it needs to be further verified in cancer models and clinical trials.

## Conclusion

A healthy stomach environment is a basis for disease prevention, while the imbalance of gastric microbiota is a potential trigger of stomach carcinogenesis. Although *H. pylori* is considered the main cause of GC, studies have shown that other gastric microbiota are also involved in the development of cancer. Therefore, we analyzed the specific interactions between *H. pylori* and non-*H. pylori* in the progression of GC and discussed effective measures to reestablish a balance stomach environment. Probiotics and dietary fiber are considered to be a preventive intervention and adjuvant treatment, while gastric microbiota transplantation may fundamentally rebuild a normal microbiota in the stomach.

In summary, the gastric microbiota is an extremely complex group. The specific mechanism that causes the occurrence and development of GC is still unclear. Gastric tumorigenesis studies should take into account the virulence diversity of *H. pylori* strains, host genetic features, entire microbiota community and diverse environmental conditions.

## Author contributions

SZ and CL conceived and designed the study, collected, and drafted the manuscript. LL, QY, and JM revised it critically for important intellectual content. WG, CD, and HW revised the manuscript. All authors contributed to the article and approved the submitted version.

## Funding

This research was funded the Fundamental Research Funds for the Central Universities (0214-14380502).

## Conflict of interest

The authors declare that the research was conducted in the absence of any commercial or financial relationships that could be construed as a potential conflict of interest.

## Publisher’s note

All claims expressed in this article are solely those of the authors and do not necessarily represent those of their affiliated organizations, or those of the publisher, the editors and the reviewers. Any product that may be evaluated in this article, or claim that may be made by its manufacturer, is not guaranteed or endorsed by the publisher.
